# Apparent Hyperandrogenemia Due to Immunoassay Interference Resolved by Liquid Chromatography–Tandem Mass Spectrometry

**DOI:** 10.1210/jcemcr/luaf140

**Published:** 2025-07-02

**Authors:** Dongni Huang, ZhiXuan Guo, JingWen Fan, Yan Zhao, Qi Pan, Lixin Guo

**Affiliations:** Department of Endocrinology, Beijing Hospital, National Center of Gerontology, Institute of Geriatric Medicine, Chinese Academy of Medical Sciences, Beijing 100730, China; Chinese Academy of Medical Sciences, Graduate School of Peking Union Medical College, Beijing 100730, China; Department of Laboratory Medicine, Beijing Hospital, National Center of Gerontology, Institute of Geriatric Medicine, Chinese Academy of Medical Sciences, Beijing 100730, China; Department of Laboratory Medicine, Beijing Hospital, National Center of Gerontology, Institute of Geriatric Medicine, Chinese Academy of Medical Sciences, Beijing 100730, China; Department of Endocrinology, Beijing Hospital, National Center of Gerontology, Institute of Geriatric Medicine, Chinese Academy of Medical Sciences, Beijing 100730, China; Chinese Academy of Medical Sciences, Graduate School of Peking Union Medical College, Beijing 100730, China; Department of Endocrinology, Beijing Hospital, National Center of Gerontology, Institute of Geriatric Medicine, Chinese Academy of Medical Sciences, Beijing 100730, China; Department of Endocrinology, Beijing Hospital, National Center of Gerontology, Institute of Geriatric Medicine, Chinese Academy of Medical Sciences, Beijing 100730, China; Chinese Academy of Medical Sciences, Graduate School of Peking Union Medical College, Beijing 100730, China

**Keywords:** testosterone, hyperandrogenemia, assay interference, liquid chromatography–tandem mass spectrometry (LC-MS/MS), diagnosis

## Abstract

Testosterone is commonly measured using immunoassays in clinical practice; however, these methods are subject to analytical interference in both men and women. In women, due to physiologically lower testosterone levels, even modest elevations may appear pathologic and complicate the diagnostic process. We report a case of a 43-year-old female patient with persistently elevated serum testosterone levels documented across multiple institutions. Despite comprehensive endocrine evaluations and imaging studies, no underlying cause could be identified. Ultimately, liquid chromatography–tandem mass spectrometry (LC-MS/MS) confirmed normal testosterone levels, indicating that the falsely elevated testosterone was due to assay interference. Further analysis using a protein precipitation assay revealed the presence of heterophilic antibodies, which were responsible for the assay interference. This case highlights the importance of considering immunoassay artifacts in the differential diagnosis of unexplained biochemical hyperandrogenism, particularly in the absence of clinical signs of androgen excess.

## Introduction

Hyperandrogenism refers to a pathological condition characterized by elevated levels or increased bioactivity of one or more androgens in the circulation, leading to gonadal dysfunction and abnormalities in energy metabolism. It is a common cause of menstrual irregularities and infertility in women. Clinical manifestations typically include oligomenorrhea or amenorrhea, hirsutism, and acne. Most cases are associated with polycystic ovary syndrome or idiopathic hirsutism. At the same time, a minority may result from more serious underlying endocrine disorders, such as adrenal or ovarian tumors, Cushing syndrome, or late-onset congenital adrenal hyperplasia [1].

Measurement of serum testosterone is essential for the diagnosis of hyperandrogenism in women. Chemiluminescent immunoassays are widely utilized in clinical laboratories due to their high efficiency and operational convenience; however, limitations such as insufficient sensitivity, lack of standardized reference materials, and cross-reactivity can lead to inaccurate results [2]. Liquid chromatography–tandem mass spectrometry (LC-MS/MS) is regarded as the gold standard for testosterone quantification, offering superior specificity and sensitivity. Nevertheless, its complexity, longer processing time, lower throughput, and limited automation have hindered its widespread clinical application [3].

In this report, we present the case of a female patient with persistent biochemical hyperandrogenism across multiple institutions, in whom extensive evaluation failed to reveal an underlying etiology. Definitive diagnosis was ultimately achieved using LC-MS/MS, which confirmed falsely elevated testosterone due to assay interference. Further investigation identified heterophilic antibodies as the source of interference.

## Case Presentation

A 43-year-old woman presented on January 14, 2025, with a 16-month history of increased menstrual volume and a 6-month history of elevated serum testosterone. Her menses had previously been regular (7-day duration, 28-day cycle) with moderate volume, bright red flow, no clots, and dysmenorrhea. Menorrhagia, noted 16 months prior, was initially ignored. Six months before the presentation, she was evaluated at an outside gynecology clinic, where hyperandrogenemia was first detected. She married at the age of 25 and had 4 pregnancies in total, including 1 live birth delivered by cesarean section 17 years ago, and 3 miscarriages. No history of androgenic medications or supplements was reported. She had a past diagnosis of prolactinoma treated with bromocriptine (1.25 mg daily), with prolactin levels consistently within normal range. On physical examination, her height was 168 cm, weight 74 kg, and body mass index (BMI) 26.22 kg/m². There were no signs of acanthosis nigricans, acne, hirsutism, voice deepening, or laryngeal prominence. No facial plethora, purple striae, central obesity, or proximal muscle weakness was noted to suggest Cushing syndrome. The gynecologic exam showed female-pattern pubic hair, normal external genitalia, and no palpable abnormalities of the uterus or adnexa.

## Diagnostic Assessment

In July 2024, serum total testosterone measured 18.411 nmol/L (0.291-1.667 nmol/L) [5.310 ng/mL] by electrochemiluminescence immunoassay (ECLIA), significantly above the reference range. Repeat testing 2 days later yielded a similar value (18.550 nmol/L). Dehydroepiandrosterone sulfate (DHEA-S) and gonadotropin levels were unremarkable, with luteinizing hormone (LH) at 17.2 IU/mL (14.0-95.6 IU/mL) and follicle-stimulating hormone (FSH) at 6.5 IU/mL (4.7-21.5 IU/mL). Other hormone levels were within normal limits (Table 1). Ultrasound showed multiple intramural uterine fibroids (largest 4.7 × 4.2 cm) and an endometrial thickness of 0.8 cm. Ovaries were visualized without abnormalities. During the same episode of care, repeat ultrasound showed stable fibroids and a right ovarian hypoechoic lesion (3.6 × 1.3 cm), which was categorized as O-RADS 5, indicating a high suspicion for malignancy according to the American College of Radiology’s Ovarian-Adnexal Reporting and Data System. Then magnetic resonance imaging (MRI) revealed: (1) multiple myometrial lesions suggestive of adenomyosis; and (2) a right adnexal ring-enhancing lesion (1.6 × 1.3 cm), likely a corpus luteum. A fluorodeoxyglucose positron emission tomography–computed tomography (FDG PET-CT) scan performed in August 2024 revealed a right adnexal isodense nodule (3.0 × 2.0 cm) with a maximum standardized uptake value (SUV) of 2.6, indicating mild FDG uptake and favoring a benign or low-grade malignant process. Testosterone remained persistently elevated throughout August and September (range, 15.450-18.330 nmol/L [4.450-5.280 ng/mL]), with a peak of 23.050 nmol/L (6.640 ng/mL) in October. However, the patient maintained regular menstrual cycles with 28- to 30-day intervals, 7-day duration, and moderate flow. Repeat imaging showed stable fibroids and a right ovarian cyst (2.9 × 2.0 cm, O-RADS 1). In January 2025, testosterone remained elevated (18.410 nmol/L). Abdominal ultrasound and adrenal-enhanced computed tomography (CT) were unremarkable. Both adrenocorticotropic hormone (ACTH) levels and cortisol diurnal rhythm were preserved. Additional laboratory tests—including 17α-hydroxyprogesterone, thyroid function, and tumor markers such as cancer antigen 125 (CA-125), cancer antigen 199 (CA-199), carcinoembryonic antigen (CEA), neuron-specific enolase (NSE), β-subunit of human chorionic gonadotropin (β-hCG), and alpha-fetoprotein (AFP)—were all within normal limits.

**Table 1.** Serial androgen and related hormone measurements with confirmatory testing

Hormone tested	Date	Result	Reference range	Assay method
Total testosterone	2024-07-19	**18.411 nmol/L (5.310 ng/mL)**	0.291-1.667 nmol/L; (0.084-0.481 ng/mL)	ECLIA (Roche Cobas e601)
	2024-07-21	**18.550 nmol/L (5.350 ng/mL)**	Same as above	ECLIA (Roche Cobas e601)
	2024-08∼09	**15.450-18.330 nmol/L (4.450-5.280 ng/mL)**	Same as above	ECLIA (Roche Cobas e601)
	2024-10	**23.050 nmol/L (6.640 ng/mL)**	Same as above	ECLIA (Roche Cobas e601)
	2025-01	**18.410 nmol/L (5.310 ng/mL)**	Same as above	ECLIA (Roche Cobas e601)
	2025-02	1.08 nmol/L (0.31 ng/mL)	0.90-3.19 nmol/L (0.26-0.92 ng/mL)	ECLIA (MAGLUMI X3)
		0.937 nmol/L (0.27 ng/mL)	0.347-2.603 nmol/L (0.10-0.75 ng/mL)	ECLIA (Beckman DXI800)
		0.833 nmol/L (0.24 ng/mL)	0.0700-1.735 nmol/L (0.02-0.50 ng/mL)	LC-MS/MS
DHEA-S	2024-07-19	0.0537 µmol/L (1.98 ng/mL)	0.027-0.159 µmol/L (1.00-5.86 ng/mL)	ELISA
LH		17.2 IU/mL	14.0-95.6 IU/mL	ELISA
FSH		6.5 IU/mL	4.7-21.5 IU/mL	ELISA
E2		181.8 pg/mL	60.4-533.0 pg/mL	CLIA
SHBG		110 nmol/L	28.3-134 nmol/L	ECLIA
ACTH, cortisol rhythm	2025-01	Normal	ND	ND
17α-hydroxyprogesterone		0.65 ng/mL	0.05-1.02 ng/mL	ECLIA
CA-125		18.6 U/mL	< 35 U/mL	ECLIA
CA-199		22.4 U/mL	< 37 U/mL	ECLIA
CEA		2.1 ng/mL	< 3.4 ng/mL	ECLIA
NSE		12.8 ng/mL	< 16.3 ng/mL	ECLIA
β-hCG		<1.0 mIU/mL	< 5 mIU/mL	ECLIA
AFP		4.5 ng/mL	< 10 ng/mL	ECLIA
FAI	2025-02	1.379%	0.827-7.072%	Calculated
SHBG		80.5 nmol/L	28.3-134.0 nmol/L	ECLIA

Abnormal values are shown in bold font. Values in parenthesis are in International System of Units (SI).

Abbreviations: ACTH, adrenocorticotropic hormone; AFP, alpha-fetoprotein; CA-125, cancer antigen 125; CA-199, cancer antigen 199; CEA, carcinoembryonic antigen; CLIA, chemiluminescence immunoassay; DHEA-S, dehydroepiandrosterone sulfate; E2, estradiol; ECLIA, electrochemiluminescence immunoassay; ELISA, enzyme-linked immunosorbent assay; FAI, free androgen index; FSH, follicle-stimulating hormone; LC-MS/MS, liquid chromatography-tandem mass spectrometry; LH, luteinizing hormone; ND, no data; NSE, neuron- specific enolase; SHBG, sex hormone–binding globulin; β-hCG, β-subunit of human chorionic gonadotropin.

## Treatment

Despite persistently elevated serum testosterone levels ranging from 17.640 to 23.050 nmol/L (5.080 to 6.640 ng/mL) across more than 10 measurements at multiple institutions—the patient maintained regular menstrual cycles and exhibited no clinical signs of androgen excess. Extensive imaging, including contrast-enhanced CT, pelvic MRI, and FDG PET-CT, revealed no ovarian or adrenal abnormalities. Although a low-dose dexamethasone suppression test is commonly used to evaluate autonomous cortisol or androgen production, it was not performed due to the absence of cushingoid features and preserved hypothalamic–pituitary–adrenal (HPA) axis function, as evidenced by normal ACTH levels and diurnal cortisol rhythm.

Upon admission to our institution, repeat hormonal testing demonstrated a serum testosterone level of 1.08 nmol/L (0.31 ng/mL) by ECLIA, with concordant free androgen index (FAI) of 1.379% (0.827%-7.072%) and sex hormone–binding globulin (SHBG) of 80.5 nmol/L (28.3-134 nmol/L) values, all within respective reference ranges. Other hormone levels were within normal limits. Repeat testing confirmed that testosterone, FAI, and SHBG all remained within reference ranges. Given the discrepancy between prior and current results, analytical interference was strongly suspected. The patient subsequently underwent additional testing using both the Beckman electrochemiluminescence assay and LC-MS/MS. In February 2025, the Beckman assay reported a testosterone concentration of 0.937 nmol/L (0.27 ng/mL), then LC-MS/MS confirmed a value of 0.833 nmol/L (0.24 ng/mL), both within normal limits. These findings supported a diagnosis of falsely elevated testosterone due to assay interference. To further validate this finding, the originating laboratory reanalyzed the patient's serum after treatment with a protein precipitation method to remove potential interferents. Post-treatment testosterone testing yielded results within the normal range, confirming the presence of assay interference.

## Outcome and Follow-Up

The patient continued to follow up with the endocrinology clinic as an outpatient. Subsequent serum testosterone measurements remained within the normal range. She was referred to the Department of Obstetrics and Gynecology, where surgical management of her uterine fibroids was planned.

## Discussion

In reproductive-aged women, approximately 25% of circulating testosterone is produced by ovarian theca cells, and 25% by the adrenal glands, and the remaining 50% is derived from peripheral conversion of precursors such as DHEA and androstenedione [4]. Thus, the differential diagnosis of hyperandrogenism can be categorized by source: ovarian (eg, polycystic ovary syndrome, ovarian stromal hyperplasia, androgen-secreting tumors), adrenal (eg, congenital adrenal hyperplasia, Cushing syndrome, adrenal tumors), and extra-glandular (eg, idiopathic hirsutism, androgenic drug use, hyperprolactinemia, stress). This patient underwent extensive evaluation at multiple institutions, with persistently elevated serum testosterone but no identified etiology. Adrenal and ovarian function tests, including 17-hydroxyprogesterone and DHEA-S, were normal, and imaging (CT, MRI, PET-CT) revealed no androgen-producing lesions. Tumor markers were unremarkable. Notably, her clinical features were inconsistent with true hyperandrogenism: she lacked hirsutism or acne, had a history of spontaneous conception, and maintained regular menstrual cycles aside from menorrhagia. Given the discordance between biochemistry and phenotype, assay interference was suspected. Testosterone levels measured by LC-MS/MS were within the normal range. Further confirmation using chemiluminescent immunoassay after protein precipitation yielded similarly normal results, supporting a diagnosis of pseudo-hyperandrogenism due to immunoassay interference.

Immunoassays commonly used to measure testosterone include radioimmunoassay (RIA), chemiluminescent immunoassay (CLIA), and enzyme-linked immunosorbent assay (ELISA). While these methods demonstrate acceptable accuracy in men, their reliability in women—whose testosterone levels are approximately 10-fold lower—is limited. Studies have shown that immunoassay-based testosterone values in women may deviate 2- to 5-fold from those obtained via LC-MS/MS [5, 6]. A comparative evaluation of 5 common automated platforms (Abbott ARCHITECT, Siemens Centaur, Beckman DXI, Roche E170, and Siemens IMMULITE) vs LC-MS/MS demonstrated that immunoassays are prone to interference and poor accuracy in low-concentration female samples [7].

Immunoassays are vulnerable to both endogenous and exogenous interference in both sexes, including cross-reactivity with serum proteins or structurally similar compounds. In women, due to lower physiological testosterone levels, even small assay-related deviations are more likely to appear abnormal and prompt clinical investigation. Among the sources of interference, heterophilic antibodies—which may arise naturally or be acquired through infection, vaccination, autoimmune disease, or exposure to animal-derived antibodies—can bind nonspecifically to assay antibodies and generate falsely elevated or decreased results [8]. This type of interference is especially associated with 2-site sandwich immunoassays, such as CLIA and ECLIA, which rely heavily on antibody-antigen interactions. Although the estimated prevalence of heterophilic antibodies in the general population ranges from 0.2% to 3.7%, studies have reported rates as high as 15% or more in hospitalized patients, likely due to increased exposure to therapeutic antibodies or other immunogenic stimuli [9].

Confirmation and mitigation of heterophilic antibody interference typically involve serial dilution, use of alternative assay platforms, application of blocking reagents, or sample pretreatment with protein-precipitating agents such as polyethylene glycol (PEG) [10, 11]. In this case, testosterone was initially measured using the Roche Cobas e601 platform and was found to be elevated approximately 10-fold. However, repeat testing on other platforms (MAGLUMI X3, Beckman DXI 800, and LC-MS/MS) yielded normal results. Following serum pretreatment with PEG, reanalysis on the Roche platform also returned normal values, thereby confirming heterophilic antibody interference. Since capture and detection antibodies differ across platforms, switching to an alternative system may reduce the risk of such interference. Additionally, PEG-based pretreatment helps remove large interfering molecules, thereby enhancing assay accuracy. LC-MS/MS remains the gold standard for testosterone quantification [12]. It separates analytes based on their physicochemical properties and quantifies them according to their mass-to-charge ratio. Compared with immunoassays, LC-MS/MS provides superior sensitivity and specificity, effectively discriminates testosterone from structurally similar steroids, and is inherently resistant to immunologic interference. Despite these advantages, its broader clinical use is limited by high cost, longer processing times (typically 4-6 hours), technical complexity, and the need for specialized personnel and instrumentation. Therefore, it is most often used to confirm questionable immunoassay results or in cases where interference is suspected.

In this case, the patient repeatedly showed markedly elevated serum testosterone levels by CLIA despite lacking clinical features of hyperandrogenism (eg, hirsutism, acne, androgenic alopecia). The discordance led to unnecessary investigations, financial burdens, and patient anxiety. Similar cases in the literature have described misdiagnosis and overtreatment—including bilateral oophorectomy or unnecessary orchiectomy—due to falsely elevated immunoassay results. These findings underscore the importance of verifying discordant testosterone elevations with LC-MS/MS to avoid misdiagnosis and inappropriate interventions.

## Learning Points

- In any case of elevated serum testosterone without clinical features of hyperandrogenism, falsely elevated results due to assay interference should be suspected and ruled out.
- Immunoassay-based testosterone measurements can be affected by heterophilic antibody interference in both men and women. However, in women, the lower normal reference range makes such interference more likely to raise clinical suspicion, potentially leading to false-positive results and unnecessary diagnostic procedures.
- LC-MS/MS confirmation is essential in cases of discrepant biochemical and clinical findings to avoid misdiagnosis and inappropriate interventions.

## Contributors

All authors made individual contributions to authorship. Lx.G., Dn.H., and Q.P. were involved in the diagnosis and management of this patient; Zx.G., Jw.F., and Y.Z. were involved in sample detection; and in addition to coordinating the manuscript submission, Dn.H. also participated in the preparation and critical revision of the manuscript. All authors reviewed and approved the final draft.

## Data Availability

Original data generated and analyzed during this study are included in this published article.

## Abbreviations

ACTH: adrenocorticotropic hormone
CLIA: chemiluminescent immunoassay
CT: computed tomography
DHEA-S: dehydroepiandrosterone sulfate
ECLIA: electrochemiluminescence immunoassay
FAI: free androgen index
FDG: fluorodeoxyglucose
LC-MS/MS: liquid chromatography–tandem mass spectrometry
MRI: magnetic resonance imaging
PEG: polyethylene glycol
PET-CT: positron emission tomography–computed tomography
SHBG: sex hormone–binding globulin
